# ERP and Behavioural Correlates of Prospective Memory in Bilinguals during L1 and L2 Processing

**DOI:** 10.3390/brainsci13020365

**Published:** 2023-02-20

**Authors:** Cristina López-Rojas, Anikó Csilinkó, Mª Teresa Bajo, Alejandra Marful

**Affiliations:** 1 Research Center for Mind, Brain and Behaviour, 18011 Granada, Spain; 2 Department of Experimental Psychology, University of Granada, 18011 Granada, Spain

**Keywords:** bilingual language processing, prospective memory, bilingualism, event-related potentials (ERPs)

## Abstract

Language influences how we process information from multiple domains. Thus, working in first (L1) or second language (L2) can modulate bilinguals’ performance on basic activities, such as visual search, decision-making, or reading. However, few studies have explored the role of L1 and L2 processing during an essential ability, such as Prospective Memory (PM). This type of memory allows us to set intentions to perform in the future (e.g., to attend an appointment). Thus, this is a novel study that allows us to explore the influence of bilingual language processing on certain cognitive abilities, which have not been deeply studied yet, such as the recall of future intentions. Thereby, this study aimed to explore the neural and behavioural correlates of bilinguals during L1 and L2 processing in a PM task where participants had to carry out an ongoing task while recovering a prospective intention given a PM cue. Importantly, the nature of the PM cue (focal or non-focal) varied the monitoring demands of the task. Behavioural and Event-Related Potential (ERP) results indicated greater engagement of monitoring processes in the PM task during L2 processing. Specifically, in L2, we found lower accuracy rates in the ongoing task and smaller amplitude differences between the focal and non-focal conditions in the P3b. Altogether, these findings suggest an impairment in prospective processing due to working in L2 contexts, supporting previous research on the impact of the bilingual experience over PM.

## 1. Introduction

Language processing is inherent in every activity that we face in our daily life. Beyond common activities, such as reading the news, having a discussion with your colleague, or writing an email, language processing is also critical when we are planning, making decisions, or generating new ideas. Research on linguistic relativity has focused on exploring how different languages modulate the way in which we think [1]. Thus, language processing influences how we integrate and interpret information from multiple domains (for a recent conceptual review see Thierry [2]). For instance, classic studies [3] showed that the way in which we categorize colours depends on the language we speak. More recently, He et al. [4] evaluated Mongolian and Chinese speakers’ colour perception and found better colour discrimination in Mongolian speakers (who have two words to describe light blue and dark blue) compared to Chinese speakers (who have only one word to describe both colours). The possible influence of language in how we process the environment is especially critical for bilingual people who have to manage the use of two or more languages when they are faced to multiple situations. Previous studies indicated that bilinguals co-activate both languages even if only one is required during the task [5,6]. This co-activation results in the need to negotiate between both languages to avoid competition when selecting the appropriate language for the context [7]. Importantly, this co-activation impacts the way in which bilingual people perceive the world and the underlying cognitive processes, especially when these activities are performed in their less dominant language [8]. Interestingly, a vast body of literature has shown the consequences of second language processing on cognitive processes, such as visual attention [9], perception of multisensory emotions [10], and long-term memory [11]. These findings demonstrate the relevance of exploring how bilingual people face daily activities in their first (L1) or second (L2) language and, more specifically, how bilingual language processing modulates specific real-world phenomena.

If we think about different tasks in day-to-day life, we can easily imagine situations in which the recall of future intentions is relevant. For example, making a call at a specific time, remembering to go to the grocery store to replenish or going to a scheduled appointment. Recalling future intentions is termed Prospective Memory (PM). To study PM in controlled experimental settings, participants are asked to perform a main activity, such as a picture naming task (i.e., ongoing task). Additionally, participants are instructed to perform an additional task (i.e., PM task) that involves the recall of a future intention in the presence of a PM cue previously encoded. For example, participants may be told that while they are performing the picture naming task (ongoing task), they have to press the key labeled in green when the picture of a ball (i.e., PM cue) appears on the screen. This event-based PM paradigm has been extensively used to study PM in the lab [12].

Cognitive processes, such as monitoring or switching [13,14], have been proposed to underlie the correct performance in this type of activity. Interestingly, the attentional processes engaged to perform the PM intention has been shown to be modulated by the type of PM cue (focal vs. non-focal cues). Thus, as stated in the Multiprocess Framework [15], properties of the focal cues engage processes that are also the main focus of the ongoing activity (e.g., a specific picture in the naming task) and can elicit the “spontaneous retrieval of the intention” without the engagement of costly monitoring or retrieval processes [16]. In contrast, when non-focal cues are presented, participants are required to pay attention to elements that are not involved in the ongoing task (e.g., detecting a specific colour framing in the screen during a picture naming task) and that signal the moment to perform the intention (i.e., PM cue). Consequently, when non-focal cues signal the PM, extra processing is required to detect this PM cue. In sum, PM allows us to create future intentions at the proper time or situation by engaging prospective processes that vary depending on the attentional demands of the task.

Similarly, bilingual people also pay attention to the environment to detect contextual cues that allow them to select the correct language in each situation [17]. Hence, this expertise in managing two languages might transfer to an essential ability, such as PM, which engages similar cognitive processes. The role of bilingualism on PM is a relevant question to be investigated since it will contribute to understanding the processes involved in both prospective memory and bilingual processing and how they modulate each other. Thereby, we describe below recent studies that address some of the relevant questions related to this topic.

In this regard, previous studies have focused on exploring the role of bilingualism as a modulator factor of performance in PM activities. For instance, López-Rojas et al. [18] studied how different bilingual experiences influence the recall of future intentions. Interestingly, they evaluated participants that differed in their bilingual experience (monolinguals, late bilinguals from a single-language context, and early bilinguals from a context with frequent language-switching). In this experiment, participants completed a non-verbal PM task that varied in their monitoring requirements (focal vs non-focal cues). In addition, online EEG brain activity was recorded during the task to explore the N300 and P3b components [19] that are related to the detection of the PM cue and to intention monitoring processes, respectively. Thus, for the N300, the early bilingual group showed an enhancement of the monitoring processes engaged to detect the PM cue during the more demanding condition (non-focal) compared to the monolinguals and late bilinguals. However, the differences associated with the bilingual experience were not evident in the less costly condition (focal). Most importantly, the P3b showed that early bilinguals adapted their monitoring strategies not only to detect the PM cues, but also during the ongoing activity to refresh the PM intention and perform it at the proper time. Nevertheless, given that the PM task in this study [18] was basically non-verbal, an intriguing question to study is how these processes vary when the PM task involves language processing and when this processing is performed in the participants’ first (L1) or second languages (L2). Thereby, López-Rojas et al. [20] aimed to explore how bilinguals’ performance in a verbal PM task varied when they completed it in L1 or L2 and how the linguistic complexity and the attentional requirements associated with the PM task varied. In a within/between subject design, they compared monolinguals’ and bilinguals’ performance in L1, but also how bilinguals differed when the task to be performed was in their L1 or in their L2. Overall, bilinguals outperformed monolinguals in L1 with faster response times and greater accuracies in the more demanding PM condition. This pattern of results suggested greater efficiency of the bilingual group in engaging the monitoring abilities required by the PM task, supporting previous studies that indicated the ability of bilinguals to adapt their cognitive strategies to the demands of the task [18,21,22,23]. Critically, in agreement with previous results in the field [24], the comparisons between L1 and L2 for bilinguals in López-Rojas et al. [20] indicated a cost associated to work in their less dominant language. In general, bilinguals showed slower response times and lower accuracies in L2 compared to L1. Again, these effects were modulated by the monitoring demands of the task so that the differences between L1 and L2 appeared in the more demanding condition (non-focal), whereas they were not evident in the less demanding focal task. Altogether, these findings indicate the critical role of bilingualism in modulating the recall of future intentions, especially when the ongoing and PM activities are performed in a second language. Unfortunately, electrophysiological data was not collected in this study, so there was no evidence of the neural correlates of the PM task in L1 and L2. These data are important given the fine-grained sensitivity of electroencephalogram information to detect the underlying cognitive processes of the bilingual experience in general cognition and, specifically, in the way in which bilingual language processing modulates certain memory processes. In this line, this experiment supposes an important contribution to the psycholinguistic field in its effort to understand how bilingualism shapes our mind and brain.

### Aims and Hypotheses

The main goal of the present experiment is to study the behavioral and electrophysiological correlations associated to first or second language processing during a PM task. To this end, our bilingual participants were asked to complete a PM task in Spanish (L1) and in English (L2). In addition, and following previous studies in the field [18,20], the nature of the PM task, i.e., focal and non-focal, was manipulated to obtain conditions that varied in their monitoring requirements [25]. This manipulation is theoretically relevant because it allows us to study the influences of the monitoring demands in the prospective processes engaged during a PM task that requires language processing.

Additionally, during the PM task, we recorded brain activity to observe the event-related potentials (ERPs) elicited by the activity. In particular, we aimed to explore the ERPs classically associated to prospective processing during a PM task: P3b and N300 [19,26,27,28]. The P3b is a positive deflection in PM trials when compared to ongoing trials over centro-parietal regions around 300–400 ms after the stimulus onset and lasting to 600–800 ms after the appearance of the stimuli. Usually, the P3b is associated to stimuli that work as targets or PM cues, reflecting working memory processes and context updating. Therefore, it is considered as signaling monitoring within the PM context [29]. On the other hand, the N300 component is a negative deflection in the occipital and parietal regions elicited by the detection of the PM cue around 300–500 ms after the stimulus appears. This component reflects a negative amplitude of the PM cues compared to the ongoing trials, which present a greater positive wave amplitude [30].

Hence, at a behavioural level, we expected to find lower performance when the task is carried out in L2 compared to L1. As previous research suggests [20], the recall of future intentions in L2 could be costlier and more demanding [31,32] and, consequently, fewer resources might be available to detect the PM cue and process the PM intention. Thus, we expected to observe slower response times and lower accuracies when the PM task is performed in L2 compared to L1. Similarly, we expected to observe the classic focality effect in our data, that is, faster response times and greater accuracies for the focal condition compared to the non-focal condition. Focality effects have been reported in a wide body of studies in the field [33,34,35], supporting the existence of dual pathways in prospective remembering [36].

In addition to the behavioural effects, we expected that L1 and L2 differences might also be reflected in the ERP components. Interestingly, to the best of our knowledge, this is the first study to explore the neural correlates associated with PM performance during first or second language processing. Based on previous results [18,19,30], we expected that the P3b and N300 components associated with differences between PM and ongoing trials would be modulated by focality and language. Thus, we expected to find a focality effect with greater amplitude-differences in the non-focal than focal conditions (that is more positive amplitude for the P3b in the non-focal than focal condition, and more negative amplitudes for the N300 in the non-focal than focal conditions). Because these focality effects reflect the capacity to adjust the monitoring and detection processes to the demands of the task, we expected that they would interact with language resulting in a lower capacity to adjust these processes when the language involved is the more demanding L2 (reflected in the ERPs with similar amplitudes for focal and non-focal conditions in L2). It is also possible, however, that L2 processing may result in larger amplitude changes in the non-focal condition relative to the focal, reflecting larger difficulties in prospective processing during the more demanding condition (non-focal) and the need to engage more cognitive resources to perform the task.

Although both hypotheses are possible, as we previously described, results comparing monolingual with early and late bilinguals [18] suggest that more difficult L2 processing might be related to impaired capacity to adjust strategies (i.e., focality effects were stronger for early bilinguals when compared with late bilinguals and monolinguals who were unsuccessful in adjusting their performance to the monitoring demands). Hence, if we assume that L2 processing will act to reduce the bilinguals’ capacity to adjust their strategies, we would expect that the effect of focality will also be reduced in L2 when compared with L1.

## 2. Materials and Methods

### 2.1. Participants

This study was approved by the Research Ethics Committee of the University of Granada (registration number, 84/CEIH/2015). A sample size of 30 was necessary to detect a medium Cohen’s d effect of 0.5 (power = 96%; α = 0.05) for a 2 × 2 repeated measures ANOVA, based on the PANGEA power analysis program [37]. A total of 31 Spanish-English bilingual volunteers (5 men; mean age = 23.06, SD = 3.425) that were university students participated in this study. All volunteers were native Spanish speakers who acquired English (L2) high fluency during childhood. In order to gain knowledge about the participants’ language experience and background, several measures were collected. Thus, the Language Experience and Proficiency Questionnaire (LEAP-Q) [38] is a validated questionnaire used to collect self-reported information about linguistic experience in the native (L1) and second language (L2), age of acquisition, frequency of language use, and exposure to each language. Additionally, to assess participants English language proficiency, the Michigan English Language Institute College Entrance Test (MELICET) was applied. The questionnaire evaluates grammar through 50 cloze questions with three answer options. Following previous studies [17,18], participants who obtained a direct score of 35 or more out of 50 were included in our experiment. Due to the fact that the MELICET was applied as a screening test, those potential participants that obtained direct scores lower than 35 did not qualify to participate in the study and were not invited to participate in the experiment. Hence, participants in the current study were preselected to reach native-like proficiency levels (M = 37.9; SD = 7.35). Table 1 reports a summary of the average scores provided by this sample to relevant items from the LEAP-Q.

### 2.2. Design

In this experiment a 2 (language: L1, L2) × 2 (focality: focal, non-focal) factorial within-subject design was employed to examine participant’s performance on the PM and ongoing (ON) tasks.

### 2.3. Procedure and Materials

All participants signed a written consent form before being evaluated. The tasks were performed in well-lit, isolated, individual rooms. The experiment consisted of two sessions in order to properly assess bilinguals’ PM performance where the PM task were carried out in their first (L1) and second (L2) language. Each experimental session lasted 90 min each, with a week in between sessions. During the first session, participants filled out the MELICET and the LEAP-Q questionnaire [38]. Then, they carried out the PM task while their brain activity was recorded using an electroencephalogram (EEG). The language of the PM task (L1 or L2) was counterbalanced across participants. During the second session participants carried out the PM tasks in the correspondent language while EEG was recorded.

#### 2.3.1. Prospective Memory Task

**Figure 1:**
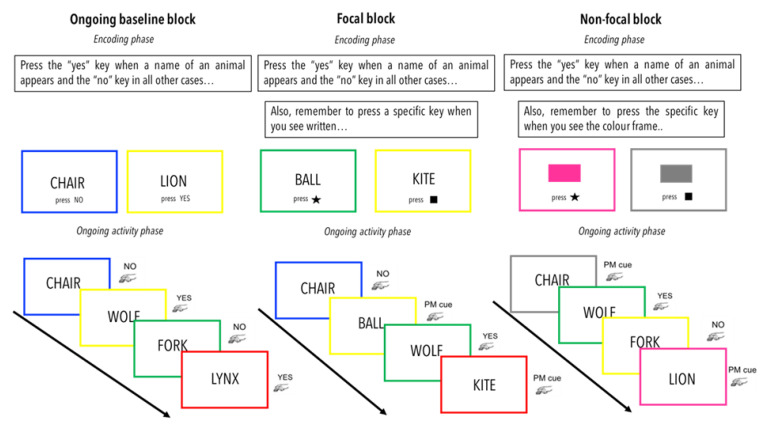
Representation of the PM task in each block condition (baseline, focal and non-focal). All the blocks started with an encoding phase succeeded by the ongoing activity alone (ongoing baseline block) or the ongoing activity with the PM intention implemented (focal and non-focal blocks). The trial sequence in each block is signaled by the black arrow. In the focal and non-focal conditions PM cues appeared intercalated.

The task consisted of three blocks of trials: baseline, focal, and non-focal. The PM task always started with the baseline block, and then, the focal or non-focal blocks occurred in a counterbalanced order. During the baseline block, participants only carried out the ongoing trial. Thus, they had to press the “yes” key when a name of an animal appeared on the screen, in any other case they were instructed to press the “no” key. Words were selected from the English Lexicon Project [39] and were controlled for length and frequency of use based on the English and Spanish corpus [40]. During the second and third block of trials, beside the ongoing task, participants were also requested to carry out the prospective intention. The cues of the prospective task were either focal or non-focal. In the focal condition participants were instructed to press “k” or “l” if the words “ball” or “kite” respectively appeared on the screen. Whereas in the non-focal condition participants were instructed to press the “k” or “l” keys when the frame bordering the screen was magenta and grey, respectively. In both conditions when the PM cues appeared, participants should interrupt the ongoing task in order to perform the prospective task. The number of trials within each block were 300 for the ongoing task and 30 for the PM task. Each word appeared until response for a maximum of 2800 ms. If participant response lasted more than 1600 ms, the following trials occurred after an inter stimuli interval (ISI) of 250 ms. When participant response was shorter than 1600 ms a black screen appeared up to 1600 ms, followed by the ISI. Presentation of the stimuli and recording of the responses were carried out on windows-based computers using E-prime 2.0 software (Psychology Software Tools, Pittsburgh, PA, USA). Figure 1 shows the trial sequence of the PM task.

#### 2.3.2. EEG Recording and Pre-Processing

Participants’ brain activity was recorded using Neuroscan Synamps2 (El Paso, TX, USA) by means of 40 Ag/AgCl electrodes allocated on the scalp. To record eye movements, two pairs of bipolar electrodes were horizontally and vertically allocated. The ground electrode was positioned in front of Fz, along the midline. The analogue EEG signal was amplified and digitised at a sampling frequency of 1000 Hz. Electrodes’ impedances were kept at <10 kΩ during recording. The EEG recording was established in an average reference. Additionally, data processing was carried out with EEGLAB 14.1 [41] using Matlab environment (Version 7.4.0, MathWorks, Natick, MA, USA). The EEG data had an online bandpass filter between 0.5–1000 Hz. Detection of channels with high levels of artefacts were identified by cautious visual inspection and interpolation method was used with neighbouring electrodes. Placement of temporal windows were following cue appearance when the stimuli were shown. The times for the ERP analysis include 200 ms pre-stimulus period used as baseline correction and 1200 ms of post-stimulus activity. Artifact correction was done using the independent component analysis toolbox (ICA) in EEGLAB with a cut of ±100 μV epoch rejection. The percentage of rejected times was always <25% for each participant after the rejection of the artefacts.

## 3. Data Analyses and Results

### 3.1. Data Analysis

#### 3.1.1. Behavioural Analyses

In our study, we analysed the response times and accuracy in the PM task following the procedure of previous studies [18,42]. Thus, accuracy scores greater than three times the interquartile range were removed from the analysis. This resulted in the removal of one participant. Additionally, individual trials with RTs faster than 200 ms were removed from the analyses. Therefore, the analyses were applied both on the PM and ON trials for each participant in each language condition (L1 and L2). Notice that only the ON trials that appeared prior to the PM cue were selected to this analysis to avoid changes due to attention fluctuation and to have the same number of ON and PM trials. Consequently, for every PM trial (30 in total) the prior ON trials (30) were included in the analysis, following a similar approach used by López-Rojas et al. [18,43]. Accordingly, 2 (language: L1, L2) × 2 (focality: focal, non-focal) repeated measures ANOVAs for the ongoing and PM were carried out. When required, post hoc tests with Bonferroni corrections for multiple comparisons were carried out. Accuracy and RTs means in the Ongoing and PM trials in both language conditions (L1 vs. L2) were reported in Table 2.

#### 3.1.2. Electrophysiological Data Analysis

Given that PM components directly compare ON vs. PM waves, a 2 (language: L1, L2) × 2 (focality: focal, non-focal) × 2 (type of trial: ON, PM) repeated measures ANOVA was carried out. Time periods were determined based on previous PM studies [18,27,30]. Therefore, we studied the P3b component connected with working memory (WM) monitoring and updating during cue detection that was registered at 300–500 ms in posterior regions [18,27] and reflects more positive amplitudes when the PM is displayed compared to the ON trials. Furthermore, we studied the N300 component, i.e., a reduction in central-posterior electrodes subsequent to the display of PM cue and relative to ON trials that is related to monitoring and (PM) cue detection. The time frame selected was between 200 to 300 ms in central-posterior regions as in previous experiments [18,43]. In addition, prior to the actual analysis, non-parametric cluster-based permutation analysis, as implemented in the Fieldtrip Matlab toolbox software [44], was performed to identify the electrodes for each time window that maximised the differences between the PM and ON trials. An advantage of this procedure is that the selection of a particular region of interest (electrode cluster) is defined in a data-driven manner and not based on the sometimes-inconsistent Regions of Interests (ROIs) from previous studies or by assumptions regarding the sampling distribution under the null hypothesis. Results of these analyses indicated that electrodes CP3, CPZ, CP4, P3, PZ, P4, O1, OZ, and O2 yielded significant differences (*p* < 0.05) for 175–300 ms intervals. For the 300–400 ms time window, the cluster included the electrodes CP3, CPZ, P3, PZ, P4, O1, OZ, and O2 (*p* < 0.05). Hence, these electrodes correspond to the usual posterior site of the N300 and to the parietal site of the P300.

After EEG data pre-processing data from five participants in L1 and one participant in L2 were eliminated due to high levels of noise in the EEG signals or a high rejection of epochs. Therefore, data was analysed with repeated measures ANOVAs of 24 participants in total.

### 3.2. Behavioural Results

#### 3.2.1. Ongoing Activity Performance

First, we analysed participants’ performance during the ongoing activity. To that end, 2 (focality: focal vs. non-focal) by 2 (language: L1 vs. L2) ANOVAs were conducted on mean response times and proportion of correct responses.

- *Response Times.* The analysis yielded a statistically significant main effect of focality condition, *F*(1,29) = 10.464; *p* < 0.05; $\eta_{p}^{2}\;$= 0.265, showing faster response times for the ongoing activity in the focal condition (*M* = 832.158, *SD* = 122.252) than in the non-focal condition (*M* = 878.308, *SD* = 154.869). However, the main effect of language *F*(1,29) = 0.247; *p* = 0.623; $\eta_{p}^{2}\;$= 0.008, and the focality by language interaction *F*(1,29) = 0.240; *p* = 0.628; $\eta_{p}^{2}\;$= 0.008 did not reach significance.
- *Accuracy.* The analysis showed a significant main effect of focality, *F*(1,29) = 87.435; *p* < 0.01; $\eta_{p}^{2}\;$= 0.751, indicating higher accuracy rates in the focal condition (*M* = 0.96, *SD* = 0.03) than in the non-focal condition (*M* = 0.93, *SD* = 0.04). Furthermore, there was a significant main effect of language, *F*(1,29) = 14.128; *p* < 0.001; $\eta_{p}^{2}\;$= 0.328, with more accuracy in L1 (*M* = 0.956; *SD* = 0.031) than in L2 (*M* = 0.934; *SD* = 0.04). However, the language by focality interaction was not significant *F*(1,29) = 0.867; *p* = 0.360; $\eta_{p}^{2}\;$= 0.029.

In sum, analyses in the ongoing activity showed a main effect of focality in response times and accuracies, indicating an impairment in the performance for the more attentional demanding conditions (i.e., non-focal). Importantly, we found a main effect of language in accuracy signalling fewer correct responses to the ongoing activity during L2 processing.

#### 3.2.2. PM Task Performance

In order to examine PM task performance, 2 (focality: focal vs. non-focal) by 2 (language: L1 vs. L2) ANOVAs were conducted on mean proportions of correct responses and response times.

- *Response Times.* The main effects of focality, *F*(1,28) = 1.951 *p* = 0.173; $\eta_{p}^{2}\;$= 0.065, language *F*(1,28) = 0.705; *p* = 0.408; $\eta_{p}^{2}\;$= 0.025, and the focality by language interaction *F*(1,28) = 3.236; *p* = 0.083; $\eta_{p}^{2}\;$= 0.104 did not reach the statistical significance.
- *Accuracy.* The main effect of focality reached statistical significance *F*(1,28) = 31.080; *p* < 0.01; $\eta_{p}^{2}\;$= 0.526, indicating greater accuracy in the focal (*M* = 0.884, *SD* = 0.149) than in the non-focal condition (*M* = 0.763, *SD* = 0.119). However, the main effect of language *F*(1,28) = 2.207; *p* = 0.149; $\eta_{p}^{2}\;$= 0.073 and the focality by language interaction were not significant *F*(1,28) = 0.833; *p* = 0.083; $\eta_{p}^{2}\;$= 0.104.

In sum, PM performance was not modulated by language condition. However, we found differences in accuracy between monitoring conditions with greater accuracy in the less demanding (i.e., focal) condition.

### 3.3. Electrophysiological Results

**Figure 2:**
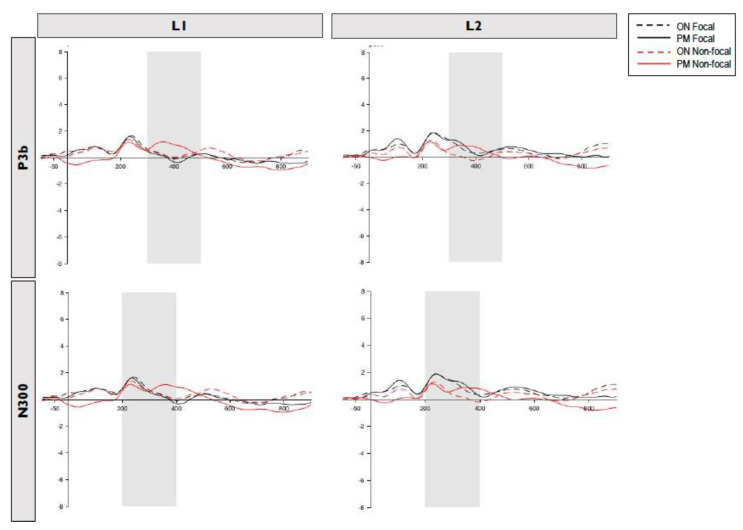
Grand-averaged event-related potentials (ERPs) at occipital-parietal electrodes indicating P3b and N300 components in function of language (L1 vs. L2) in the focal and non-focal condition of the ON and PM trials.

For each ERP component (P3b and N300), mean amplitudes across electrodes and conditions were averaged and adhered to a 2 (language) × 2 (focality) × 2 (type of trial) within measures ANOVA. Figure 2 shows the P3b and N300 components in function of language (L1 vs. L2) in the focal and non-focal condition in the PM and ON trials.

#### 3.3.1. Electrophysiological Results: P3b

Although the main effects of focality *F*(1,23) = 0.007; *p* = 0.935; $\eta_{p}^{2}\;$= 0.000, and language *F*(1,23) = 0.123; *p* = 0.729; $\eta_{p}^{2}\;$= 0.005 did not reach statistical significance, the main effect of type of trial *F*(1,23) = 4.519; *p* = 0.044; $\eta_{p}^{2}\;$= 0.164 was significant, indicating greater positive amplitude in PM trials (*M* = 0.555; *SD* = 1.03) compared to ON trials (*M* = 0.321; *SD* = 1.18). Interestingly, the focality by type of trial interaction reached statistical significance *F*(1,23) = 8.179; *p* = 0.009; $\eta_{p}^{2}\;$= 0.262, indicating that there were no significant differences between ON (*M* = 0.465; *SD* = 1.39) and PM trials (*M* = 0.423; *SD* = 1.16) (t(24) = 0.303, *p* = 0.764, d = 0.03) in the focal condition. However, in the non-focal condition the differences between type of trials reached significance (ON: *M* = 0.177; *SD* = 0.97; PM: *M* = 0.686; *SD* = 0.91; t(24) = −3.305, *p* < 0.05, d = −2.03). Thus, the standard P3b appeared in the non-focal condition but not in the focal condition. Additionally, the focality by language interaction *F*(1,23) = 4.518; *p* = 0.044; $\eta_{p}^{2}\;$= 0.164 revealed that the focality effect associated to P3b appeared in L1 t(24) = −1.910, *p* = 0.068, d = −0.34 (i.e., greater positivity in non-focal *M* = 0.593; *SD* = 0.777 than in the focal condition *M* = 0.278; *SD* = 1.109) but it was not evident in L2 (focal, *M* = 0.742; *SD* = 1.350; non-focal *M* = 0.350; *SD* = 0.745) t(28) = 1.623, *p* = 0.116, d = 0.33. Nevertheless, the interactions of language by type of trial *F*(1,23) = 0.195; *p* = 0.663; $\eta_{p}^{2}\;$= 0.008, focality by language by type of trial *F*(1,23) = 0.249; *p* = 0.623; $\eta_{p}^{2}\;$= 0.011 were not significant.

In sum, the P3b component (higher amplitudes in the PM when compared to the ON) appeared in the non-focal condition, where more involvement of monitoring processes were expected. Interestingly, the differences between monitoring conditions disappeared when the task was performed in L2, suggesting a general impairment in the available cognitive resources to face the task during second language processing.

#### 3.3.2. Electrophysiological Results: N300

The main effect of focality was significant *F*(1,23) = 10.282; *p* = 0.004; $\eta_{p}^{2}\;$= 0.309 with more negative amplitudes in the non-focal condition (*M* = 1.088; *SD* = 1.51) compared to the focal condition (*M* = 1.66; *SD* = 2.02). This indicated greater engagement of monitoring processes to detect the non-focal PM cue. None of the remaining main effects or interactions reached statistical significance (type of trial *F*(1,23) = 1.900; *p* = 0.181; $\eta_{p}^{2}\;$= 0.076; language condition *F*(1,23) = 0.002; *p* = 0.967; $\eta_{p}^{2}\;$= 0.000; focality by type of trial interaction *F*(1,23) = 1.214; *p* = 0.282; $\eta_{p}^{2}\;$= 0.050; focality by language *F*(1,23) = 2.400; *p* = 0.135; $\eta_{p}^{2}\;$= 0.094; type of trial by language *F*(1,23) = 0.092; *p* = 0.765; $\eta_{p}^{2}\;$= 0.004; focality by type of trial by language *F*(1,23) = 0.164; *p* = 0.689; $\eta_{p}^{2}\;$= 0.007).

The N300 analyses indicated a lack of differences between ON and PM trial processing, suggesting the same engagement of detection processes for both types of trials. However, we found that the N300 was modulated by the monitoring condition. We will go back to this result in the next section.

## 4. Discussion

In the current study, we aimed to shed light on the impact of bilingualism over the recall of future intentions. Particularly, we focused on examining behavioural and neural correlates associated to focal and non-focal PM tasks when they were performed in the first or second language of bilingual’s participants. Thus, bilingual participants completed a PM task that varied in its monitoring (focal and non-focal conditions) and language requirements (i.e., L1 and L2). During the task, participants’ brain activity (EEG) was recorded to be able to identify the prospective processes that may be affected by the language used in the PM task.

Behavioural data from the ongoing task clearly yielded significant differences between focality conditions, showing faster response times and higher accuracy rates for the ongoing activity (ON) in the focal condition compared to the non-focal condition. This better performance for the focal condition when compared to the non-focal condition was also observed for the PM task with a significant effect in accuracy. This pattern of results is in agreement with the Multiprocess Framework for Prospective Memory [15] in suggesting that “spontaneous recovery” of the PM intention is more likely to occur when focal cues are presented, resulting, thus, in higher accuracy and faster RTs for focal than non-focal cues, which usually require monitoring processes (i.e., the so-called focality effect; [14,30]). Thus, our results support previous findings in the field, which observed focality effects in different populations [34,43].

Most importantly, participants reached lower accuracy rates in the L2 condition than in the L1 when performing the ongoing task. This pattern of results supports previous findings exploring the impact of the bilingual experience over a PM task [18]. Specifically, the impairment in performance when the ongoing activity was in the participants’ less dominant language is consistent with previous findings by López-Rojas et al. [23] that found lower ongoing performance in bilinguals that worked in their L2. Similar to the present study, this impairment was independent of the monitoring demands of the task. Altogether, these results indicated that the processing of a second language requires the engagement of cognitive resources [24] and, as a result, fewer resources may be available to prospective processing during the ongoing task. Interestingly, this pattern of data is in agreement with a vast body of literature on L2 reading comprehension which showed lower performance when reading in a foreign language (for a revision see Melby-Lervåg & Lervåg [45]). Similarly, Pérez et al. [24] found an impairment in the high cognitive processes engaged during inferential text revision in L2. Therefore, this study adds new evidence on the impact of second language processing during a memory task.

Nevertheless, performance in the PM task (detection of the cue and execution of the intention) was not influenced by language and showed similar focality effects for L2 and L1, a result that differed from López-Rojas et al. [20] where stronger focality effects were found in L2 than in L1. This different pattern of results in the PM task can be due to the higher linguistic demands in López-Rojas et al. [20], where the PM cues were embedded in a highly demanding sentence comprehension task, when compared to the current study, where the PM consisted in identifying a frame colour or a given word. Hence, we suggest that future research might design more linguistically complex PM tasks to measure the fine-grained behavioural effects of recalling a future intention in L2.

Interestingly, the impairment of prospective processing during second language processing were indicated by the ERP analysis. First, an analysis of the P3b evidenced a main effect of type of trial, showing greater positive amplitude in the PM trials compared to the ongoing trials. This finding is in line with the results observed in the behavioural data, namely PM trials engaged monitoring processes related to cue detection. In addition, we observed focality effects in the non-focal condition that were not evident in the focal condition. These patterns of results suggest that WM and context updating may be more strongly involved when the nature of the ON cue elicits monitoring and not spontaneous retrieval as is the case of non-focal cues [27,30]. This pattern is consistent with previous results in López-Rojas et al., [18] where, in the more difficult non-focal condition, early bilinguals showed larger differences between ON and PM trials compared to late bilinguals and monolinguals, whereas these differences were not present in the focal condition. Thus, they suggested that early bilinguals engaged in monitoring processes related to prospective processing to adapt to the task’s demands. Therefore, our results suggest that PM can be modulated depending on the bilingual experience. In consequence, these data are consistent with results from previous studies indicating that bilingual experience shapes our brains and modulate cognitive processes such as monitoring or switching (see Antoniou [46]).

Most importantly, in the current study the interaction focality by language yielded significant results showing that the focality effect associated to P3b appears in L1, with greater positivity in the non-focal condition, compared to the focal condition. However, in L2, these differences between focal and non-focal were not significant. These patterns of results suggest that while bilinguals successfully modulated their strategies to adjust to the task’s demands in L1, this is not possible in the L2 probably due to the higher cognitive load in the second language. Therefore, consistent with López-Rojas et al. [18], in L1 participants successfully engaged in updating and monitoring processes with the aim to adjust their strategies to the demands of the task in the more demanding non-focal condition. By contrast, when immersed in L2, the higher attentional load might possibly impair the monitoring processes required for prospective remembering. These results are in line with previous results indicating that an enhancement in conflict resolution when the task was performed in a bilingual context was related to a reduction in the P3b [47]. In summary, the present pattern of data suggests that L2 processing reduced the participants’ capacity to adjust their PM strategies to the demands of the task so that the differences between focal and non-focal conditions were no-longer present.

However, results for the N300 were puzzling. Although the typical focality effect was evidenced by the data and non-focal cues produced more negative amplitudes than focal cues (the usual effect of type of trial (ON vs. PM)), the effect of language or their interactions were not significant. The lack of differences between PM and ON trials is surprising since the N300 component is characterized by a negative deflection triggered by the PM trials in comparison to the ON trials [19], signaling the engagement of prospective processes during PM cue detection. Notice, however, that the N300 has not always been detected in some PM studies [19,48,49,50], which points to the elusive nature of this component.

The fact that we found a significant focality effect independently of the type of trial suggests that the component captured by our analysis in the time window from 200 to 300 ms might be better characterized as an N200 (for a characterization see Patel & Azzam [51]) than as an N300. Several studies have explored the role of N200 in different memory tasks [52,53]. Specifically, in PM tasks this component reflects more negative amplitudes over posterior regions around 200–350 ms [54] in conditions where the ongoing trial is concurrent with a PM intention when compared to baseline conditions where the ongoing trials are not accompanied by the PM intention [55,56]. The interpretation of this effect as an N200 component would be consistent with our current pattern of data: a focality effect with greater negativity in the non-focal condition where extra monitoring is required to detect the PM cue and absence of differences between ON and PM trials. Moreover, the N200 has been characterized as a neural correlate of monitoring during visual detection [52] with more negative amplitudes with perceptual cues [56]. Thereby, it might be possible that our pattern of results indicated greater engagement of monitoring processes during the non-focal condition due to the perceptual nature of the PM cue and the need of checking the colour frame to detect the PM trials. Thus, although this interpretation needs further research, both the more perceptual nature of the PM cue and the higher monitoring demands in the non-focal condition support the presence of a more negative N200 component in this condition when compared to the focal condition.

In sum, the present study supports previous findings in the field about the role of bilingual experience and linguistic contexts on the processes that underlie PM performance [20]. Hereby, we showed the impact of language processing on an ability such as prospective processing. PM plays a fundamental role in daily activities, and failures may result in dramatic consequences (i.e., forgetting to turn off the oven). Hence, studies exploring whether and how the cognitive mechanisms underlying PM can be modulated by the use of different languages are critical. Our findings highlight the importance of the linguistic context where we encode and recall future intentions, signalling possible impairments in PM when the task involves L2 processing. Behavioural and ERP data support this suggestion with worse performance in L2 ongoing activity and the absence of focality effects in P3b during L2 processing. To the best of our knowledge, this study is the first to examine the neural correlates associated to PM tasks during first and second language processing. Future research should explore the impact of language processing during a more linguistically complex PM task that resembles the rich linguistic context in which bilinguals are immersed in their daily activities (e.g., a text comprehension task). In addition, future studies should explore the role of the linguistic context during the encoding and recall of future intentions, and how language-incongruent encoding and recall of PM intentions can modulate performance. Finally, it is important to remark on the applied relevance of this study in the educational field or in the professional area, especially if we think about bilingual environments where bilingual people encode and execute future intentions in their second language. Given this, future studies should deepen in the relationship between linguistic context and memory processes.

## 5. Conclusions

This study highlights the importance of working in a first or second language during the retrieval of future intentions (PM task). Interestingly, we found distinct impacts of language over the ongoing and PM activity, suggesting that L2 processing produce a general impairment in performing PM tasks. Our behavioural and neural (P3b) results suggest that processing a second language could impose a load on the available cognitive resources and, as a result, fewer resources remain available to face the main activity. Altogether, this experiment raises interesting conclusions about the role of language processing in daily activities, such as the recall of future intentions, and opens up new venues for inquiry.

## Figures and Tables

**Table 1:** Mean score and standard deviations in L1 and L2 questions from the LEAP-Q.

	L1	L2
Proportion of current exposure to the language	0.64 * (0.16)	0.30 * (0.14)
Proportion of preference to read in each language	0.51 * (0.23)	0.43 * (0.21)
Proportion of preference to speak in each language	0.54 * (0.29)	0.35 * (0.22)
Mean age of beginning acquisition (years)		4.71 (2.66)
Mean age of becoming fluent (years)		12.87 (4.94)
Mean level of self-competence (from 0–10)		8.5 (1.26)
Mean level of language exposure with family or friends (from 0–10)		3.87 (3.25)
Mean level of reading exposure (from 0–10)		5.93 (3.69)
Mean level of language exposure by TV or radio (from 0–10)		8.58 (2.19)
Mean level of language exposure by self-learning (from 0–10)		2.54 (3.29)

* Indicated significant differences (*p* < 0.05) between L1 and L2 comparisons.

**Table 2:** Means ACC and RTs (with standard deviation in parentheses) by type of trial and language session in PM task.

	L1		L2	
Type of Trial	ACC	RT	ACC	RT
ON trials focal	0.97 (0.03)	836 (142)	0.95 (0.03)	828 (102)
ON trials non focal	0.94 (0.03)	889 (184)	0.92 (0.04)	868 (126)
PM trials focal	0.85 (0.19)	1059 (256)	0.92 (0.1)	1002 (199)
PM trials non focal	0.75 (0.14)	1069 (206)	0.78 (0.1)	1071 (189)

## Data Availability

The data that support the findings of this study are available from the corresponding author upon request.
